# DNA Repair Pathway Alterations in Bladder Cancer

**DOI:** 10.3390/cancers9040028

**Published:** 2017-03-27

**Authors:** Kent W. Mouw

**Affiliations:** Department of Radiation Oncology, Dana-Farber Cancer Institute/Brigham & Women’s Hospital, Harvard Medical School, Boston, MA 02215, USA; kent_mouw@dfci.harvard.edu; Tel.: +1-617-852-9356

**Keywords:** urothelial cancer, bladder cancer, DNA repair, nucleotide excision repair, mutational signature, genomic instability

## Abstract

Most bladder tumors have complex genomes characterized by a high mutation burden as well as frequent copy number alterations and chromosomal rearrangements. Alterations in DNA repair pathways—including the double-strand break (DSB) and nucleotide excision repair (NER) pathways—are present in bladder tumors and may contribute to genomic instability and drive the tumor phenotype. DNA damaging such as cisplatin, mitomycin C, and radiation are commonly used in the treatment of muscle-invasive or metastatic bladder cancer, and several recent studies have linked specific DNA repair pathway defects with sensitivity to DNA damaging-based therapy. In addition, tumor DNA repair defects have important implications for use of immunotherapy and other targeted agents in bladder cancer. Therefore, efforts to further understand the landscape of DNA repair alterations in bladder cancer will be critical in advancing treatment for bladder cancer. This review summarizes the current understanding of the role of DNA repair pathway alterations in bladder tumor biology and response to therapy.

## 1. Introduction

### 1.1. Bladder Cancer Is a Global Health Problem

An estimated 75,000 cases of bladder cancer will be diagnosed in the USA in 2017, making it the fifth most common cancer among adults [1]. Worldwide, it is predicted that nearly 500,000 cases are diagnosed annually, although rates vary significantly across geographical regions [2]. Several environmental risk factors—including cigarette smoke, occupational exposures, and infectious agents—have been identified [3]. In addition, genetic factors may also play a role.

In the USA, the majority of bladder cancer cases are diagnosed at an early stage and can be managed with cystoscopic tumor removal followed by close surveillance. However, nearly one in four patients will present with muscle-invasive bladder cancer (MIBC), which requires aggressive multimodality therapy to provide an opportunity for cure. Approximately 5% of patients present with metastatic disease at diagnosis, and outcomes are poor in this population, with an average survival of approximately one year despite aggressive therapy.

The majority of bladder tumors are urothelial (transitional cell) carcinomas. Although early-stage (non-MIBC) can demonstrate low- or high-grade histologic features, nearly all MIBCs are high-grade. Less common histologic variants also occur, and can be associated with unique clinical features and genetic properties that are reviewed elsewhere [4,5].

### 1.2. DNA Repair Pathway Alterations Are Biomarkers and Therapeutic Targets

Genomic instability is a hallmark of tumors, and alterations in DNA repair pathways can drive tumor behavior and response to treatment [6,7]. Recent evidence from numerous large-scale sequencing efforts has demonstrated that alterations in DNA repair pathways are common across cancer types [8]. For example, germline and somatic alterations in the homologous recombination (HR) pathway have long been known to play a role in the development and clinical progression of many breast and ovarian tumors [9]; however, it is now apparent that HR pathway dysfunction—driven by alterations in *BRCA1*, *BRCA2*, or other HR genes—also plays an important role in other settings such as prostate, gastric, and endometrial cancers [10].

Numerous chemotherapeutic classes—including platinum compounds and alkylating agents—exploit the relative DNA repair deficiency of tumors and continue to play a critical role in the treatment of many tumor types [11]. In addition, the discovery and recent clinical approval of poly (ADP ribose)polymerase (PARP) inhibitors in several HR-deficient tumor settings is an example of the potential to target DNA repair-deficient tumors using a synthetic lethal approach [12]. Numerous clinical trials are now underway to test the safety and efficacy of other DNA repair targeted agents in a variety of DNA repair-deficient and proficient tumor settings [13]. 

## 2. The Genomic Landscape of Bladder Cancer Reveals Alterations in DNA Repair Pathways

### 2.1. Bladder Tumors Exhibit a Complex Somatic Mutational Landscape

In 2014, the Cancer Genome Atlas (TCGA) published a comprehensive genomic analysis of 131 high-grade MIBCs [14]. The tumors had a large number of genomic alterations, with an average of more than 300 exonic mutations, 200 copy-number alterations (gain or loss), and 20 chromosomal rearrangements per tumor. In total, recurrent mutations were identified in 32 genes, with *TP53* being the most commonly mutated gene (in 49% of samples). Similarly, *CDKN2A* was focally deleted in nearly 50% of cases. Several chromatin-modifying genes were significantly mutated, including *KDM6A*, *MLL2*, and *ARID1A.* Translocations involving *FGFR3* or *ERBB2* were each observed in multiple cases, and *ERBB2* was also frequently mutated. Taken together, the somatic landscape of MIBC revealed alterations in cell cycle and/or chromatin remodeling pathways in the majority of tumors as well as potentially targetable alterations in the *PIK3A*/*AKT*/*MTOR* and receptor tyrosine kinases (RTKs) such as *FGFR3*, *EGFR*, and *ERBB2*.

**Figure 1:**
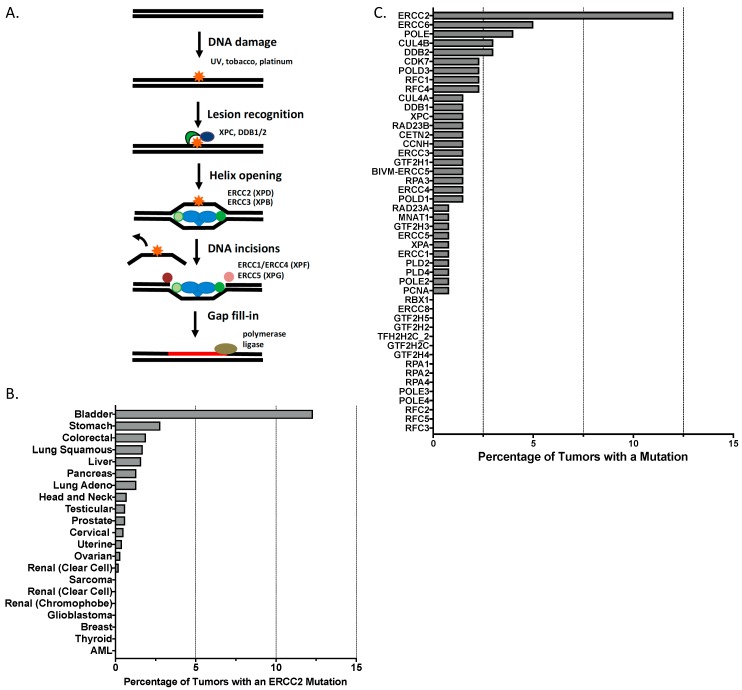
Nucleotide excision repair (NER) pathway mutations in bladder cancer and other tumor types. (**A**) Schematic overview of the NER pathway with select genes of interest highlighted. (**B**) *ERCC2* somatic mutation frequency across published or provisional TCGA tumor cohorts. (**C**) Frequency of somatic missense mutations in NER pathway genes in TCGA bladder cohort.

Although alterations in chromatin remodeling, the cell cycle, and the RTK pathway can impact DNA repair function in tumors, relatively few ‘classic’ DNA repair genes were among the genes that were significantly mutated in the TCGA cohort. One interesting exception was *ERCC2*, a core member of the nucleotide excision repair (NER) pathway, with 12% of the cohort harboring somatic *ERCC2* missense mutations (Figure 1). Several tumors had truncating mutations in *BRCA2* or *ATM*, but neither of these genes reached statistical significance across the cohort. 

*NFE2L2*, which encodes the transcription factor Nrf2, was mutated in 8% of TCGA tumors. Nrf2 drives expression of genes in the oxidative response pathway, and *NFEL2L* mutations frequently disrupt binding to KEAP1, which normally targets Nrf2 for degradation [15]. *NFEL2L2* mutations were mutually exclusive with mutations in the redox regulator *TXNIP* (mutated in 7% of tumors), and were associated with a distinct transcriptional profile. Given that many DNA damaging agents commonly used in bladder cancer (including gemcitabine and 5-fluorouracil) induce oxidative stress, alterations in genes involved in oxidative stress response pathways may have important implications for chemotherapy response [16].

### 2.2. Mutational Signatures Reflect Underlying DNA Damage and Repair Processes

Despite the relative paucity of somatic alterations in DNA repair genes compared to tumor types such as breast or ovarian cancer, the impact of DNA damage and repair processes in bladder cancer is evident from its mutational landscape. Like other carcinogen-induced tumors, the somatic mutation burden in bladder tumors is quite high, with a median of nearly eight mutations per megabase [14]. Indeed, the only common tumor types with higher average point mutation burdens are melanoma and non-small cell lung cancer [8].

In addition to overall mutation burden, the pattern of mutational changes in a tumor can provide insight regarding the underlying DNA damage exposure and repair processes that have been active in a tumor over time. Individual mutational processes are frequently associated with a specific pattern of base changes—a so-called “mutational signature”—that can be inferred from the overall mutational spectrum. Several mutational signature analyses have been performed across bladder cancer cohorts and have provided important insights regarding the DNA damage and repair landscape.

Alexandrov and colleagues published the first mutational signature analysis of bladder cancer and identified four distinct signatures [17]. One of these signatures (COSMIC database signature 1) has been identified as a “clock-like” signature that is characterized by C > T transitions at CpG motifs resulting from spontaneous deamination of 5-methylcytosine [18]. Two signatures (COSMIC database signatures 2 and 13) are characterized by frequent C > T and C > G mutations and are thought to arise following APOBEC-mediated cytosine deamination [19]. Finally, a signature characterized by a broad spectrum of base changes was identified (COSMIC data signature 5). Although an etiology had not previously been proposed for signature 5, subsequent work has revealed that signature 5 is enriched among tumors with somatic *ERCC2* mutations [20]. Across three separate bladder cancer cohorts, *ERCC2* was the only gene for which mutation status was significantly associated with signature 5 activity, suggesting that the spectrum of point mutations comprising signature 5 may represent the mutational effects of loss of normal NER function. Furthermore, signature 5 activity was also associated with tobacco exposure, which is known to create DNA adducts that are typically repaired by the NER pathway. Together, these analyses suggest that APOBEC activity and NER loss may contribute to the mutational spectra observed in MIBC, and more broadly, demonstrate the ability of mutational signature analyses to reveal mutational processes that shape the tumor genome.

### 2.3. Gene Expression Profiles Define Distinct MIBC Subtypes

In addition to DNA-based approaches, TCGA and other groups have performed comprehensive transcriptomic analysis of MIBCs and have identified several biological subtypes based on differences in gene expression patterns. These subtypes often have distinct histopathological features and are associated with unique clinical behavior and response to treatment (discussed below) [21].

RNA-seq analysis of TCGA tumors identified four MIBC expression subtypes: clusters I and II had expression profiles that shared features with luminal breast cancers while clusters III and IV had prominent expression of mesenchymal cell markers resembling basal-like breast tumors and some squamous tumors. Similarly, several groups have applied microarray-based gene expression profiling to large MIBC cohorts, and although the specific names given to different subtypes vary, the separation of tumors into luminal and basal subgroups was evident across studies [22,23,24,25]. Based on observed differences in numerous gene sets—including genes involved in cell cycle regulation, epithelial-mesenchymal transition (“stemness”), and immune cell infiltrate—it is reasonable to assume that important differences in DNA repair function exist across subtypes, although these differences remain to be fully characterized.

## 3. DNA Repair Pathways Are Predictive Biomarkers in Bladder Cancer

DNA damaging agents play a critical role in the treatment of MIBC. The addition of neoadjuvant cisplatin-based chemotherapy prior to cystectomy improves overall survival compared to cystectomy alone [26,27]. Trimodality therapy (TMT), which consists of maximal transurethral tumor resection (TUR) followed by concurrent chemoradiotherapy, is an alternative to cystectomy and provides an opportunity for patients to maintain a functional bladder [28,29]. Given that the success of both approaches depends on the therapeutic efficacy of DNA damaging agents, there is significant interest in identifying predictive biomarkers to help guide the use of these agents in MIBC.

### 3.1. Low ERCC1 Expression Is Associated with Improved Outcomes in Cisplatin-Treated Patients

Cisplatin is used in the treatment of a variety of solid tumors and directly damages cellular DNA through formation of intra- and inter-strand platinum-DNA crosslinks [30]. The nucleotide excision repair (NER) pathway is a highly conserved DNA repair pathway that efficiently detects bulky adducts such as those formed by platinum drugs and is capable of repairing these lesions in an error-free manner (Figure 1A) [31]. Given the close link between cisplatin-mediated DNA damage and NER-mediated repair, numerous studies have investigated the association between NER pathway activity and cisplatin response in a variety of tumor settings.

One of the first reports of an association between NER activity and clinical cisplatin response in bladder cancer was published by Bellmunt and colleagues in 2007 (Table 1) [32]. In the study, reverse transcriptase (RT)-PCR was used to measure mRNA levels of several DNA repair genes in a cohort of advanced bladder cancer patients treated with cisplatin-based chemotherapy. Median survival was significantly longer in patients with tumors that had low expression levels of *ERCC1*, an NER gene that forms a heterodimeric nuclease with ERCC4 and is responsible for cleaving the DNA backbone adjacent to the damaged base. Low levels of ERCC1 expression were hypothesized to reflect decreased NER capacity and thus explain the increased tumor sensitivity to cisplatin-mediated DNA damage and improved clinical outcomes among cisplatin-treated bladder cancer patients. The association between ERCC1 protein expression (measured by immunohistochemistry (IHC)) and survival following cisplatin-based therapy was recently validated in a separate cohort of metastatic bladder cancer patients [33]. Interestingly, similar IHC-based studies in post-cystectomy or chemoradiotherapy contexts have reported better outcomes in cases with high ERCC1 staining [34,35]. It is possible that ERCC1 status may have different prognostic and predictive implications across clinical contexts, or that differences in technique or reagents may contribute to differences in staining patterns when using IHC-based approaches. More broadly, an association between *ERCC1* expression and cisplatin response has also been reported in other tumor types, although many of these studies lack prospective validation or have suffered from difficulties with reproducibility [36,37,38,39].

### 3.2. High MRE11A Expression Is Associated with Improved Outcomes in Radiation-Treated Patients

Differences in expression levels of genes in DNA repair pathways beyond NER have also been associated with bladder tumor response to DNA damaging agents. The homologous recombination (HR) pathway plays a critical role in the repair of double-strand breaks (DSBs) resulting from damage caused by ionizing radiation, inter-strand cross-linkers such as cisplatin and mitomycin C, and intercalating agents such as doxorubicin. Although *BRCA1* and *BRCA2* alterations are relatively common in some tumors settings and have important implications for treatment selection, somatic alterations or expression changes (resulting from *BRCA1* promoter methylation or other epigenetic events) are relatively uncommon in bladder tumors [14].

In an attempt to determine if changes in other DSB repair genes were associated with treatment response in MIBC, Choudhury and colleagues used an IHC-based approach to measure levels of five DSB repair proteins (MRE11, ATM, RAD50, NBS1, and H2AX) in pre-treatment biopsies from bladder cancer patients treated with radiation [40]. In the test cohort, patients with tumors that had MRE11 expression in the lowest quartile had significantly worse cause-specific survival (CSS) compared to patients with higher MRE11 expression (three-year CSS, 43% vs. 69%) (Table 1). This association was validated in a second radiation cohort; however, there was no association between MRE11 levels and CSS in a third cohort of patients who were treated with cystectomy rather than radiation. Similar findings have now been reported by several other groups. Lauberg et al. reported significantly worse disease-specific survival (DSS) among low-expressing MRE11 tumors treated with radiation but no difference in DSS based on MRE11 expression levels among cystectomy patients [41]. Most recently, analysis of MIBC patients treated across six Radiation Therapy Oncology Group (RTOG) cooperative groups trials again demonstrated an association between low levels of MRE11 expression and increased disease-specific mortality (four-year DSM, 41% for lowest quartile of MRE11 staining vs. 21% for remainder of cohort) [42].

The association between MRE11 and radiation response in these studies is perhaps counter-intuitive, as it could be anticipated that decreased expression of a DSB repair gene such as MRE11 would be associated with decreased repair capacity and thus increased radiation sensitivity. However, the opposite effect was observed: lower MRE11A levels were associated with worse outcomes in each study. It is possible that low MRE11A levels may fail to trigger a DNA damage response sufficient to signal growth arrest and thus allow tumor cells to continue to proliferate despite the presence of unrepaired DNA damage; however, additional studies are needed to understand the molecular underpinnings of this association. It is interesting that in the TCGA bladder tumor cohort, transcript levels of *MRE11A* do not vary significantly and somatic events (mutations or epigenetic silencing) are rare, suggesting that cellular MRE11A protein levels may be controlled post-transcriptionally [48].

### 3.3. Basal-like Tumors Benefit Most from Chemotherapy

As discussed previously, works by multiple groups have used gene expression profiling to identify and characterize several distinct MIBC subtypes. Similar to breast cancers, MIBCs can be broadly categorized into luminal and basal subtypes [49]. In addition to having distinct clinical and histopathologic features, MIBC expression subtypes also carry unique prognostic information. Similar to breast cancer, basal-like MIBCs have a worse prognosis and are associated with significantly shorter disease-specific and overall survival among MIBC patients treated with upfront cystectomy [22,23]. However, in addition to being prognostic, expression subtype appears also to be predictive of response to treatment. Despite a worse prognosis in the absence of chemotherapy, the addition of neoadjuvant cisplatin-based chemotherapy (NAC) prior to cystectomy improved outcomes among patients with basal-like tumors such that the overall survival of these patients was significantly better than patients with luminal or “p53-like” tumors who also received NAC [50]. Patients with p53-like tumors (a subset of luminal tumors) had the poorest response to NAC, and furthermore, comparison of matched pre- and post-NAC tumors demonstrated that post-NAC tumors of all subtypes adopt a p53-like expression profile [22,50]. The p53-like expression profile is characterized by a low expression of late cell cycle markers, suggesting that tumor cells may become chemoresistant by entering a quiescent-like state.

Taken together, these findings underscore the ability of expression profiling to provide important prognostic and predictive information. As evidenced by their differential sensitivity to DNA damaging regimens, MIBC subtypes harbor important differences in DNA damage signaling and repair capacity. Unraveling the mechanisms that drive differences in DNA repair capacity across histopathologic and expression-based tumor subtypes could have important implications for the management of bladder cancer as well as other tumor types with similar transcriptional subtypes.

### 3.4. Somatic ERCC2 Mutations Are Associated with Improved Outcomes among Cisplatin-Treated Patients

Although the use of cisplatin-based chemotherapy is associated with an overall survival benefit in large randomized studies, tumor response varies widely across patients. Given an uncertain benefit in the face of the known cisplatin-related toxicities, only a fraction of eligible MIBC patients receive cisplatin-based regimens [51]. Thus, reliable predictive biomarkers are needed to identify the patients most likely to benefit from cisplatin-based treatment.

In an effort to identify somatic alterations associated with cisplatin response in MIBC, Van Allen and colleagues performed whole exome sequencing of tumor and matched germline DNA of a cohort of 50 MIBC patients [43]. All patients were treated with neoadjuvant cisplatin-based chemotherapy followed by cystectomy, and the cohort was selected to include 25 “responders” with no evidence of invasive disease (pT0/pTis) on pathologic examination of the cystectomy specimen and 25 “non-responders” with residual muscle-invasive disease (≥pT2). Four genes were significantly mutated across the cohort of 50 patients—*TP53, RB1, KDM6A,* and *ARID1A*—all of which have been implicated in MIBC biology. In addition, the authors performed an enrichment analysis to identify genes that were more frequently mutated in responders compared to non-responders. This analysis yielded only one significant gene—*ERCC2*. Nine of 25 responders (36%) had a somatic missense mutation in *ERCC2* compared to 0 of 25 non-responders (Table 1).

*ERCC2* encodes an NER helicase that unwinds the DNA duplex adjacent to sites of damage caused by genotoxins such as UV irradiation and platinum drugs (Figure 1) [52]. Biallelic germline *ERCC2* mutations can result in xeroderma pigmentosum, an autosomal recessive disease characterized by extreme UV sensitivity [53]. Given the critical role of the NER pathway in repairing DNA damage caused by platinum compounds, it was hypothesized that the observed somatic *ERCC2* mutations drive cisplatin sensitivity by conferring loss of normal cellular NER function. Indeed, functional analysis of a subset of the mutants revealed that they were unable to rescue the cisplatin or UV sensitivity of an *ERCC2*-deficient cell line.

Recently, the association between somatic *ERCC2* mutations and cisplatin sensitivity was validated in a separate MIBC cohort [44]. Eight of 20 cisplatin responders (40%) harbored an *ERCC2* mutation versus only two of 28 non-responders (7%). In addition, in both the discovery and validation cohorts, patients with *ERCC2*-mutated tumors who received cisplatin-based neoadjuvant chemotherapy lived significantly longer than patients with WT *ERCC2* tumors. A similar survival difference between mutant and WT *ERCC2* cases was not observed in the TCGA cohort—a cohort in which no patients received neoadjuvant chemotherapy. Thus, *ERCC2* mutations may be predictive of cisplatin response rather than simply prognostic of better outcomes in the absence of chemotherapy.

Somatic *ERCC2* missense mutations were present in approximately 15% of tumors in the TCGA cohort. Interestingly, bladder cancer is the only solid tumor type identified to date in which *ERCC2* is significantly mutated (Figure 1B). Conversely, no other genes in the NER pathway appear to be frequently mutated in bladder cancer (Figure 1C). Therefore, a unique relationship seems to exist between *ERCC2* mutations and bladder cancer tumorigenesis, and additional work will be needed to define the molecular role of *ERCC2* mutations as a bladder cancer driver.

### 3.5. Mutations in DNA Repair Genes beyond ERCC2 Are also Associated with Improved Outcomes

In addition to *ERCC2*, somatic alterations in other DNA damage and repair genes have been associated with improved response to DNA damage-based therapy in bladder cancer (Table 1). For example, Plimack and colleagues performed targeted sequencing of 287 cancer-related genes in two cohorts of patients treated with neoadjuvant cisplatin-based chemotherapy and cystectomy. Decision tree analysis revealed that in both discovery and validation cohorts, patients with a somatic mutation in one or more of the DNA repair genes *ATM*, *FANCC*, and *RB1* were significantly more likely to have no residual muscle-invasive disease at cystectomy [46]. Of note, *ERCC2* was not included in the initial panel of cancer genes, but was subsequently sequenced and used to validate the association between somatic *ERCC2* mutations and response (as reported by Liu et al. [44] and described above). 

In a similar study reported by researchers from the University of Chicago, whole exome or targeted sequencing of tumors from MIBC patients receiving perioperative chemotherapy revealed improved recurrence-free survival among patients with tumors harboring a mutation in one or more of six DNA repair genes (*ATM, ERCC2, FANCD2, PALB2, BRCA1,* and *BRCA2*) [47]. Similarly, alterations in one or more of a panel of 34 DNA repair genes were associated with improved survival among patients with locally advanced or metastatic bladder cancer receiving first-line platinum-based chemotherapy [54]. Finally, DNA repair gene mutations were also associated with a trend towards decreased disease recurrence among MIBC patients treated with primary chemoradiotherapy [45]. In the cohort, somatic *ERCC2* mutations were significantly associated with reduced metastatic recurrence rate, but no association between MRE11 protein expression levels and outcomes was noted.

Taken together, these studies provide compelling evidence for an interaction between DNA repair gene alterations and treatment response in bladder cancer patients receiving DNA damaging-based therapy. However, in these studies, events in multiple DNA repair genes (often representing more than one repair pathway) were considered together, so the impact of individual genes (beyond *ERCC2*) on treatment response remains to be determined. In addition, as the number of queried genes in a “DNA repair gene” list grows, the event rate (i.e., the likelihood of a mutation in at least one gene in the list) may begin to reflect overall tumor mutation burden rather than gene- or pathway-specific processes (i.e., the mutations may be passenger rather than driver events), and tumor mutation burden has also been associated with response to DNA damaging agents in several of these (and other) studies [43,46]. Therefore, additional work is needed to more fully understand the complex interaction among specific DNA repair alterations, global genomic instability, and response to DNA damaging-based therapies such as platinum agents and radiation.

### 3.6. Germline DNA Repair Polymorphisms Are Associated with Bladder Cancer Risk and Treatment Response

In addition to the well-known associations linking DNA damaging agents such as cigarette smoke and analine dyes with bladder cancer risk, attempts have also been made to identify germline DNA repair alterations that impact the risk of developing bladder cancer and/or predict response to treatment. Lynch syndrome is characterized by inherited mutations in one of the mismatch repair (MMR) genes (*MLH1, MSH2, MSH6*, or *PMS2*) and is associated with an increased risk of multiple cancer types [55]. Although Lynch syndrome patients are known to have an increased risk of developing upper tract urothelial tumors, a recent Dutch study also found an increased risk of bladder tumors among Lynch syndrome patients, with a relative risk of 4.2 for men and 2.2 for women [56]. This association was confirmed in a Canadian cohort [57]. In both cohorts, the risk of bladder cancer appeared to be highest among *MSH2* mutation carriers. However, germline alterations in other well-known DNA repair genes such as *BRCA1, BRCA2*, or *ATM* have not been clearly linked to bladder cancer risk.

Germline determinants of bladder tumor response to systemic therapy have also been investigated [58]. Although single nucleotide polymorphisms (SNPs) in several genes, including DNA repair genes such as *XPA* and *ERCC2*, have been associated with improved response, none of these markers have been rigorously validated [59,60]. Several SNPs in *MRE11A* were associated with worse outcomes in a radiation cohort, but not a cystectomy cohort [61]. Given previous reports from the same group linking tumor MRE11 expression levels with radiation outcomes, this germline association is compelling, but requires validation in an independent radiation-treated cohort.

## 4. Ongoing Efforts to Optimize Bladder Cancer Treatment

With the remarkable amount of genomic information now available from sequencing- and expression-based studies, an important challenge for the field will be to use genomic insights to guide translational research efforts and ultimately to inform clinical trial design in bladder cancer. Many cancer centers are now incorporating tumor genomic testing (including DNA- and/or RNA-based analyses) into routine clinical management, although significant logistical challenges regarding timing, payment, and interpretation remain [62].

The impact of DNA repair pathway alterations on sensitivity to DNA damaging agents has been demonstrated in several retrospective analyses (discussed above) and is also supported by preclinical data. However, in order to shift clinical practice, prospective data are needed. To this end, several prospective trials are planned that stratify MIBC patients by mutation status of one or more DNA repair genes. Given that the presence of a DNA repair gene mutation is associated with improved response to DNA damaging-based treatment, it is exciting to think that treatment decisions could be tailored based on the DNA repair features of a tumor. In this regard, MIBC patients with tumor DNA repair alterations may be ideal candidates for organ-sparing approaches that utilize DNA damaging-based chemotherapy and radiation.

In addition to increasing sensitivity to DNA damaging agents such as cisplatin, loss of DNA repair capacity can also be associated with sensitivity to DNA repair targeted therapies. PARP inhibitors are the most well-developed DNA repair targeted agents and are now approved in several DNA repair deficient clinical settings [12]. However, unlike breast and ovarian tumors, in which the vast majority of DNA repair alterations involve the homologous recombination pathway, bladder tumors have alterations across several DNA repair pathways, including higher rates of NER pathway alterations than other tumor types (i.e., *ERCC1* and *ERCC2*; Figure 1). Although the PARP1 enzyme is recruited to sites of UV damage and interacts with the NER protein DDB2 to promote repair [63,64], PARP inhibitors are synthetic lethal only in the setting of tumor HR deficiency. Therefore, the fraction of bladder tumors sensitive to PARP inhibitors, particularly as monotherapy, may be modest. Preclinical data demonstrating activity of other DNA repair targeted agents in bladder cancer have been reported [65,66,67], and a trial investigating the addition of an ATR kinase inhibitor to cisplatin and gemcitabine in patients with metastatic urothelial cancer was recently launched (NCT02567409).

One of the most exciting advances in bladder cancer is the recent approval of several immune checkpoint inhibitors (ICIs) for treatment of advanced bladder cancer patients. In the past year, three different agents have been approved for use in patients with metastatic disease, including in both cisplatin-naïve and cisplatin-refractory settings. ICIs agents block inhibitory immune signaling molecules, allowing the patient’s immune system to mount a more effective anti-tumor immune response. Although response to ICIs varies widely within and across tumor types, the most robust activity has been observed in carcinogen-associated tumors with high mutation burdens such as bladder cancer, non-small cell lung cancer, and melanoma. Somatic point mutations resulting from carcinogen-induced damage or from an underlying tumor DNA repair deficiency can lead to production of neoantigenic peptides which are recognized as non-self by the host immune system. Although there is a correlation between somatic mutation burden and ICI sensitivity across tumor types, the relationship among somatic mutation burden, tumor neoantigens, host immune activation, and ICI response is clearly multifaceted and is the subject of intense ongoing study.

The first ICI approved for use in bladder cancer was the anti-PD-L1 agent atezoluzimab, and exploratory biomarker analyses from the Phase 2 trial revealed associations between TCGA expression subtype and response rate as well as between mutation load and response rate [68]. Although responses were observed across expression subtypes, the highest response rate was observed among luminal cluster II tumors, which have transcriptional profiles consistent with activated effector T cells. Interestingly, the basal expression subtypes (clusters III and IV) also have an activated T cell expression signature as well as high rates of PD-L1 staining on tumor and immune cells, but had lower response rates to atezoluzimab, suggesting that additional immune-suppressive signals are active in these tumors. In addition, mutation burden (measured across 315 sequenced genes) was positively correlated with atezoluzimab response: the median mutation burden was nearly twice as high in responders compared to non-responders.

These findings underscore the impressive activity of ICIs in bladder cancer and also highlight the importance of integrating clinical, pathologic, and genomic biomarkers to provide the best opportunity to guide clinical ICI use. Numerous additional trials are ongoing, and it is very likely that the scope of ICIs in bladder cancer will expand in the coming years. As ICIs are incorporated earlier in treatment regimens, one important challenge that is particularly relevant to bladder cancer will be to understand the interplay between ICIs and DNA damaging agents. Although DNA damage can lead to somatic mutations that ultimately result in tumor-specific neoantigens, DNA damaging agents can also have immune suppressive effects that may dampen the anti-tumor immune response. These interactions are complex and multi-faceted, and further clinical and pre-clinical work is needed. It is possible that the optimal integration of immune checkpoint blockade with DNA damaging agents may vary significantly across tumor types and clinical contexts. 

DNA repair pathway alterations have the potential to be important biomarkers and therapeutic targets in bladder cancer, and additional studies may reveal that the true scope of DNA repair pathway dysfunction in bladder cancer is even broader than is currently appreciated. Integrative approaches that combine genomic, proteomic, and functional approaches will be required to more fully understand the contribution of DNA repair dysfunction to bladder tumor biology and treatment response. 

## Figures and Tables

**Table 1:** Clinical implications of select DNA repair pathway alterations in bladder cancer.

Gene	Pathway	Cohort/Study Details	References
*MRE11A*	DSB repair	Low MRE11 staining associated with worse survival following RT for MIBC; no association between MRE11 levels and survival in cystectomy patients	[40,41,42]
*ERCC1*	NER	Low *ERCC1* mRNA levels associated with improved survival in advanced/metastatic BC patients treated with cisplatin-based chemotherapy	[32]
		High nuclear ERCC1 staining associated with worse survival in metastatic BC patients treated with cisplatin-based chemotherapy	[33]
*ERCC2*	NER	Somatic *ERCC2* missense mutations associated with improved pathologic response and survival in MIBC patients receiving neoadjuvant cisplatin-based chemotherapy followed by cystectomy	[43,44]
		Somatic *ERCC2* missense mutations associated with decreased metastatic recurrence rate in MIBC patients receiving chemoradiotherapy	[45]
>1 gene	DSB repair, others	Alteration(s) in *ATM*, *RB1*, or *FANCC* associated with improved pathologic response in MIBC patients receiving neoadjuvant cisplatin-based chemotherapy followed by cystectomy	[46]
		Alteration(s) in *ATM, ERCC2, FANCD2, PALB2, BRCA1*, or *BRCA2* associated with increased RFS in MIBC patients treated with cystectomy and peri-operative chemotherapy	[47]
		Alteration(s) in ≥1 of 20 DDR genes associated with trend towards decreased recurrence in MIBC patients treated with chemoradiotherapy	[45]

RT: radiation therapy; MIBC: muscle-invasive bladder cancer; BC: bladder cancer; RFS: recurrence-free survival; DDR: DNA damage response.
